# Interruption of of enteral nutrition in the intensive care unit: a single center survey

**DOI:** 10.1186/2197-425X-3-S1-A289

**Published:** 2015-10-01

**Authors:** M Uozumi, M Sanui, T Komuro, Y Iizuka, T Kamio, H Koyama, H Mouri, T Masuyama, K Ono

**Affiliations:** Emergency and Critical Care Medicine, Dokkyo Medical University, Utsunomiya-shi, Japan; Anesthesiology and Critical Care Medicine, Jichi Medical University Saitama Medical Center, Saitama, Japan

## Introduction

Interruption of enteral nutrition (EN) is common in the intensive care unit (ICU). Frequent episodes of EN interruption can lead to protein and calorie debt with a potential impact on outcomes in critically ill patients [1, 2]. However, few studies have investigated the details of EN interruption practice including the reasons and duration of the interruption [3, 4]. A standard protocol to minimize EN interruption has not been established.

## Objectives

In this retrospective single center survey, the current practice of EN interruption was investigated to support future development of an interruption-minimizing protocol.

## Methods

This is a review of 100 patients in the ICU more than 72 hours and receiving EN in a 12-bed, medical/surgical ICU in a tertiary care center between January and December 2013. Data were collected from the electronic medical record including patient demographic data; total time designated for EN; the number of EN interruption episodes; reason of each interruption episode categorized as: diagnostic examination, therapeutic intervention, and gastrointestinal (GI) events, and their individual subcategories; duration of each interruption episode; and presence of written orders for interruption.

## Results

The median ICU length of stay was 17.1 (interquartile range [IQR] 3- 71) days. Mean APACHE II score was 21.8 (standard deviation 6.8). Crude hospital mortality was 19%. The total number of EN interruption episodes in all patients was 571. Of these, 515 (89%) EN interruption episodes were in intubated patients. There were a mean of 8.5 episodes per patient. EN interruption was performed for unknown reasons (166 episodes, 29.1%), GI events (78 episodes, 14.7%) and hemodialysis (71 episodes, 12.4%). The median duration of EN interruption in all patients was 9.47 (0.5 - 203) hours. The cumulative time of episodes corresponds to 19% of the total time designated for EN. The duration of EN interruption, varied by reason including extubation (15 [1 - 53] hours), tracheostomy (9.5 [1 - 24] hours), and possible intubation (6.5 [1.5 - 68] hours). Only sixty episodes (11.7%) had written orders for interruption.

## Conclusions

According to this single-center study, interruption episodes during EN in the ICU are frequent, the reason and duration of the interruption varied, and airway procedures had relatively longer durations of interruption. Documentation and orders were frequently missing. These results warrant development of a protocol for EN interruption.
